# Can NLR, PLR, and LMR be used as prognostic indicators in patients with pulmonary embolism? A commentary

**DOI:** 10.17305/bjbms.2020.5236

**Published:** 2021-08

**Authors:** Cihan Bedel, Mustafa Korkut, Hamit Hakan Armağan

**Affiliations:** 1 Department of Emergency Medicine, Health Science University Antalya Training and Research Hospital, Antalya, Turkey; 2 Department of Emergency Medicine, Faculty of Medicine, Suleyman Demirel University, Isparta, Turkey

We read with great interest the article “Prognostic role of neutrophil-lymphocyte ratio (NLR), platelet-lymphocyte ratio (PLR), and lymphocyte-monocyte ratio (LMR) in patients with pulmonary embolism” by Köse et al. [1]. They found that the NLR, PLR, and LMR were related to the prognosis and clinical severity of patients with pulmonary embolism (PE). First of all, we congratulate the authors for their invaluable contribution to literature. However, we think that there are some points that should be discussed regarding the use of these laboratory parameters.

White blood cell subtypes NLR, PLR, and LMR have been associated with many inflammatory diseases, including PE [2,3]. These parameters, which can be easily determined by simple and easy measurement of systemic inflammation, maintain their importance today. However, these parameters are affected by many factors such as trauma, local or systemic infection, acute coronary syndromes, and malignancy [3-5]. For these reasons, it would be better for the authors to mention these factors and exclude them from the tables that included malignancy and trauma patients in the study.

It is known that drugs, including steroids, can increase neutrophils and decrease lymphocytes and therefore affect NLR, PLR, and LMR values [6]. Therefore, it will be more valuable to exclude patients who use drugs that may affect laboratory parameters. In addition, plasma inflammatory biomarkers are time-dependent variables, the time of sample collection and the time from the onset of the symptom to the sampling may have an impact on the parameters [3-6]. Therefore, it is important to identify the time from the first symptom to sample collection and the factors that may affect it. In conclusion, because NLR, PLR, and LMR can be affected by many factors, prospective studies with large populations are needed to show the accuracy of use in critically ill patients.
